# Asymmetric biphasic electric stimulation supports cardiac maturation and functionality

**DOI:** 10.1177/20417314251393556

**Published:** 2025-11-28

**Authors:** Antonio Sileo, Stefano Gabetti, Alp Can Gülan, Igor Cervenka, Chunyan Zhang, Alma Mingels, Giulia Milan, Diana Massai, Anna Marsano

**Affiliations:** 1 Department of Surgery and Department of Biomedicine, University Hospital Basel, University of Basel, Basel, Switzerland; 2 Department of Mechanical and Aerospace Engineering and PolitoBIOMed Lab, Politecnico di Torino, Torino, Italy; 3 Department of Biomedicine, University Hospital Basel, University of Basel, Basel, Switzerland; 4 Department of Clinical Chemistry, Central Diagnostic Laboratory, Maastricht University Medical Center Maastricht, Maastricht, The Netherlands

**Keywords:** electrical stimulation, monophasic waveform, biphasic waveform, asymmetric biphasic waveform, cardiac tissue engineering

## Abstract

Two-dimensional (2D) cardiac models are widely used for cardiotoxicity screening but often lack structural and functional maturity of adult native tissue. Electrical stimulation (ES) enhances *in vitro* maturation, yet conventional waveforms (monophasic and symmetric biphasic) have shown limitations, including charge accumulation and possible cell hyperpolarization. Here, we introduce for the first time an asymmetric biphasic ES waveform that combines the advantages of monophasic and symmetric biphasic stimulation by reversing the current and reducing residual voltage. Asymmetric biphasic stimulation improved electrical functionality, calcium handling and contractility of neonatal rat cardiac cells, without triggering cellular stress. Additionally, cells subjected to asymmetric biphasic ES displayed a metabolic shift toward fatty acid oxidation, a hallmark of mature cardiomyocytes. Taken together, these findings highlight the novelty and efficacy of asymmetric biphasic stimulation in generating more physiologically relevant *in vitro* cardiac models, providing a promising alternative to standard ES protocols.

## Introduction

*In vitro* two-dimensional (2D) cardiac models are commonly used to assess drug-induced cardiotoxicity, safety and efficacy, due to their simplicity, cost-effectiveness, and compatibility with high-throughput screening.^1–3^ These models typically employ monolayers of cardiac cells to evaluate the effects of pharmacological compounds on contractility, and electrophysiological properties beside cell viability. However, conventional 2D cardiac cultures often rely on immature cell types, such as neonatal rat cardiomyocytes or human-induced pluripotent stem cell-derived cardiomyocytes, which lack the structural and functional characteristics of adult human cardiomyocytes.^1,4^ Moreover, limited cell-cell and cell-matrix interactions in these systems hinder the development of mature sarcomeric architecture, calcium handling, and contractile activity.^5,6^ Improving cardiac structural and functional maturation of cardiomyocytes is therefore crucial to enhance the accuracy and predictive value of *in vitro* cardiac models for both pharmacological screening and basic biological studies. Several studies underscore the need for more physiologically relevant cardiomyocyte models to improve the assessment of electrophysiological and mechanical toxicity testing, as well as to investigate fundamental cardiac biology.^7,8^ To address these limitations, various strategies have been explored, including topographical,^9–12^ biochemical,^9,13^ mechanical,^14–20^ and electrical cues,^21–31^ as well as three-dimensional (3D) culture systems.^8,32–34^ Among these approaches, electrical stimulation (ES) has emerged as an effective strategy to enhance functional coupling, contraction strength, and electrophysiological properties leading to improved synchronicity, conduction velocity, and overall contractile performance.^21–23,35,36^ ES is commonly delivered via electrodes submerged in culture medium,^37^ and the application of monophasic pulse has been demonstrated to improve cell alignment, sarcomere organization, and electrophysiological activity.^24,25^ However, electrochemical reactions, specifically nonreversible Faradaic reaction, at the electrode–electrolyte interface occur, which can lead to electrode degradation, local pH imbalances,^26,27^ and the accumulation of potentially cytotoxic by-products in the medium.^38,39^ Biphasic ES has therefore been proposed as an alternative to mitigate charge build-up. This stimulation mode consists of two half waves of opposite polarity: the first triggers cardiomyocyte contraction, while the second, with inverted polarity, reverses the chemical processes induced by the first wave.^27,28^ Furthermore, preliminary studies have shown that symmetric biphasic ES (in which both phases have equal amplitude) can enhance cardiac cell maturation compared to monophasic ES, improving excitability, connectivity, and sarcomeric organization.^29–31^ However, some evidence suggests that its secondary negative phase may hyperpolarize cardiomyocyte membranes, potentially interfering with subsequent action potential generation.^28^ Thus, achieving a balance between stimulation efficacy and biocompatibility remains a key challenge.

Here, we introduce for the first time an asymmetric biphasic ES mode, composed of two phases of different amplitudes. This novel approach has been designed to combine the respective advantages of monophasic and biphasic stimulation, while minimizing their inherent limitations. We hypothesized that asymmetric biphasic ES can promote functional cardiomyocyte contractions, while reducing cell damage potentially caused by by-products released during ES. Using our custom-built electrical stimulator (ELETTRA^31^), capable of delivering multiple, customizable ES waveforms in parallel, we systematically compared asymmetric biphasic ES to monophasic and symmetric biphasic ES in 2D culture. Additionally, asymmetric biphasic ES was evaluated against the conventional monophasic mode in a 3D culture system.

## Materials & methods

### Electrical stimulation

#### Tunable electrical stimulator interfaced with parallel culture chambers

A tunable ES setup, previously developed,^31^ was used to deliver different electrical waveforms to 2D and 3D cell cultures. Briefly, the custom-made setup is based on (i) the ELETTRA electrical stimulator, designed for *in vitro* ES of cardiac cells and tissues, and (ii) parallel, transparent-bottom culture chambers. ELETTRA is a compact device, based on an Arduino Due micro-controller board (Arduino, Italy), providing voltage-controlled monophasic or biphasic square wave pulses, adjustable in voltage (0.25–12 V), frequency (0.5–10 Hz), and pulse duration (1–10 ms). It enables controlling three independent output channels, each of which can be connected to multiple culture chambers. The chambers consist of 35 mm μ-Dishes (Ibidi GmbH, Germany) equipped with an autoclavable polydimethylsiloxane (PDMS, Sylgard 184, Dow Corning, United States) insert featuring a central rectangular well. Two parallel carbon rod electrodes (length = 26 mm, diameter = 3 mm; Sigma-Aldrich, Germany) are embedded within the insert at a fixed distance of 1 cm and are connected to ELETTRA via platinum wires (diameter = 0.3 mm; Polyfil AG, Switzerland). Carbon was selected among different electrode materials due to its properties of biocompatibility, charge transfer, and corrosion resistance.^31,40^ Each electrode is exposed to the culture medium along a 20 mm segment. Within each culture chamber, a uniform electrical field develops at the bottom and in the central portion, ensuring uniform stimulation of cell monolayers and 3D constructs.^31^

#### Selection of ES modes

The ES modes were designed to minimize residual voltage at the end of each stimulation pulse and to optimize the total charge delivered to the cultured cardiomyocytes. An equivalent lumped-parameter model of the culture chamber and of the ELETTRA waveform generation unit was used to simulate the system behavior and to characterize voltage and current waveforms (Simulink, MathWorks, United States). The culture chamber was modeled as a Simplified Randles Cell circuit,^39,41–43^ characterized by resistance of the solution (*R_e_*) equal to 53 Ω, resistance of the electrodes to corrosion (*R_p_*) equal to 5.13 × 10^8^ MΩ, and non-ideal capacitance of double layer at the electrode/electrolyte interface (*C_p_*) equal to 240 μF, with *R_p_* and *C_p_* in parallel (for details on adopted assumptions see Supplemental Material). The simulations were run considering 4 chambers connected in parallel to one ELETTRA output and applying different ES modes. Exploiting the full waveform tunability of ELETTRA, three distinct ES modes were selected: (1) Monophasic (Mono) – pulses with electric field amplitude of 3 V/cm and pulse duration of 2 ms, at a frequency of 1 Hz; (2) Symmetric biphasic (Sym Bi) – pulses at ±1.5 V/cm, with the same pulse duration and frequency; (3) Asymmetric biphasic (Asym Bi) – pulses with electric field amplitude of +3 V/cm in the positive half-wave and −1 V/cm in the negative half-wave, with the same pulse duration and frequency.

The Mono ES mode was selected as the reference condition for evaluating two alternative strategies aimed at mitigating its known limitations.^26,27,38,39^ The Sym Bi ES mode was selected because the reversal of electric field polarity can reduce electrode degradation by discharging the electrodes through current inversion, resulting in minimal residual voltage. Compared to the Mono ES at 3 V/cm mode, the Sym Bi ES at ±1.5 V/cm delivers the same absolute value of the electric field variation, as the positive and negative phases each provide half the field strength (Supplemental Figure S1), and half the amount of charge during the stimulation. To combine the advantages of both the Mono and the Sym Bi modes, the Asym Bi mode (+3/−1 V/cm) was specifically designed to: (1) deliver, during the positive half-wave, comparable peak stimulation intensity to Mono ES while reducing overall exposure time; (2) apply, during the negative half wave, an electric field of opposite polarity to facilitate electrode discharge (Supplemental Figure S1) and minimize charge accumulation. During the positive phase, Asym Bi ES delivers the same electric field amplitude as the Mono ES mode (3 V/cm), but for half of the pulse duration. During the negative phase, Asym Bi applies an electric field of opposite polarity using a lower amplitude than the positive phase to reduce the total charge delivered compared to the Mono ES. To select the amplitude of the negative phase, simulations were run using the lumped-parameter model and the final value of −1 V/cm for the negative phase was decided based on the estimated residual voltage.

#### Characterization of ES modes

Experimental tests were performed to evaluate the residual voltage, the total delivered charge and the total energy for each ES mode and to verify the compliance with the lumped parameter model outcomes. Four culture chambers were filled with 2.5 ml of DMEM and were connected in parallel to ELETTRA. The Mono ES, the Sym Bi ES, and the Asym Bi ES modes were delivered. The current was monitored using a digital oscilloscope (PicoScope 2204A, Pico Technologies, United Kindom), connecting the probe to the ELETTRA monitoring ports and 10 independent recordings were acquired for each of the voltage waveforms were recorded at a sample rate of 780 kS/s. The acquired signals were post-processed using MATLAB R2021b (MathWorks, United States) to evaluate the residual voltage, the total delivered charge and the total energy. Mean and standard deviation were calculated for each quantity.

### Cell culture systems

#### Cell isolation

Neonatal rat cardiac cells were isolated from 2 to 3-day-old Sprague Dawley rats following established protocols.^44^ Briefly, rat ventricles were minced into small pieces and digested overnight at 4°C in a 0.06% w/v trypsin solution (trypsin from bovine pancreas, Sigma-Aldrich, USA) with continuous shaking at 50–60 oscillations per minute. The digestion was continued with five consecutive 4-min cycles using a 0.1% w/v collagenase solution (type 2 collagenase, Worthington-Biochem, USA). To improve the cardiomyocyte fraction, the isolated cardiac cells were pre-plated in culture flasks for 45 min at 37°C and 5% CO₂ to allow fibroblasts to attach. The enriched cardiac cell population (>70% cardiomyocytes) was then seeded at a density of 6 × 10^4^ cells/cm^2^ and cultured for 48 h before starting the experiments. For the first 24 h, the cells were maintained in high glucose (HG) Dulbecco’s Modified Eagle’s Medium (DMEM, Sigma-Aldrich, USA), supplemented with 1% v/v HEPES buffer (Sigma-Aldrich, USA), 1% v/v penicillin/streptomycin (Sigma-Aldrich, USA), 1% v/v L-glutamine (Sigma-Aldrich, USA), and 10% v/v fetal bovine serum (FBS, Sigma-Aldrich, USA; seeding medium). During the last 24 h, the cells were maintained in low glucose (LG) DMEM (Sigma-Aldrich, USA), supplemented with 1% v/v HEPES buffer (Sigma-Aldrich, USA), 1% v/v penicillin/streptomycin (Sigma-Aldrich, USA), 1% v/v L-glutamine (Sigma-Aldrich, USA), and 1% v/v FBS (Sigma-Aldrich, USA) to limit the proliferation of cardiac fibroblasts (culture medium).

#### 2D cell culture under ES

Before starting the experiments, the culture chambers were prepared as previously described.^31^ Briefly, in each culture chamber the PDMS structures equipped with the carbon rod electrodes were press-fit. Cells were then seeded at a density of 6 × 10^4^ cells/cm^2^, equivalent to 2.5 × 10^5^ cells per chamber, using 2 mL of seeding medium. The following day, the seeding medium was replaced with 2.5 mL of culture medium. For each condition, four samples were statically pre-cultured for 3 days to allow the cells to recover from the isolation process. This 3-day pre-culture period, without ES, was chosen based on previous studies^39,45,46^ to facilitate recovery of neonatal rat cardiac cells post-isolation. Subsequently, cardiac cells were cultured for an additional 4 days either without (Control) or with ES, to evaluate the short-term effects of ES as reported in earlier studies.^23,29–31,47^ The culture medium was refreshed every 2 days to supply fresh nutrients and remove toxic by-products generated during ES.

**Figure 1.**
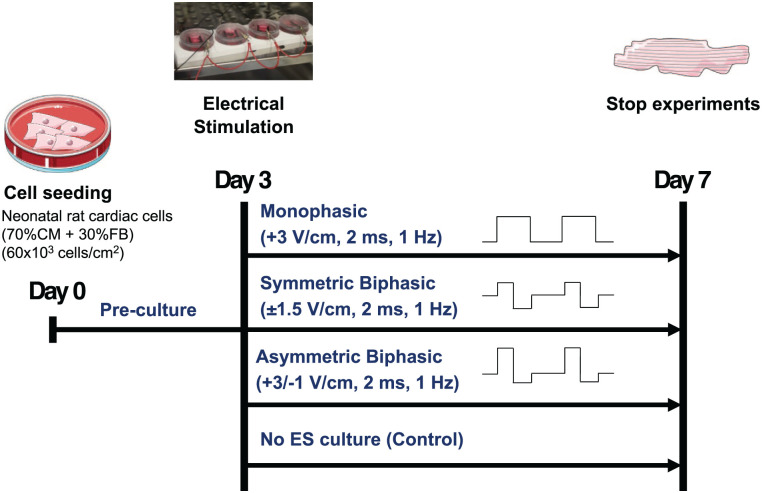
Experimental setup of the 2D cardiac culture model.

Three different ES modes were tested simultaneously, using a frequency of 1 Hz, and a pulse duration of 2 ms: Monophasic ES at 3 V/cm (Mono), Asymmetric biphasic ES at +3/−1 V/cm (Asym Bi), Biphasic ES at ±1.5 V/cm (Sym Bi; Figure 1).

ES modes were applied in parallel to four culture chambers, and all samples were cultured for a total of 7 days in a standard incubator (37°C, 95% humidity, 5% CO₂; Figure 1).

#### 3D cell culture under ES

Traditional 2D cardiac cell models are limited in their capacity to mimic the native heart’s 3D architecture and the dynamic interactions with the extracellular matrix (ECM). To better replicate these features, 3D culture approaches incorporating fibrin hydrogels have been introduced. Fibrin gel forms through the enzymatic reaction of fibrinogen and thrombin, followed by calcium crosslinking catalyzed by activated Factor XIII, creating a stable network that embeds cells. Fibrin is preferred in cardiac tissue engineering, compared to the collagen, due to its rapid polymerization,^48^ ECM-supportive environment,^49^ and mechanical flexibility.^50^ However, its tendency to degrade over time is a drawback.^49^ To address this, tranexamic acid is added to prevents fibrinolysis by inhibiting the conversion of plasminogen into plasmin.^51^

Hydrogel-based engineered cardiac tissues (ECTs) were generated by seeding rat origin cardiac cells in a 100 mL fibrin gel solution (25 mg/mL fibrinogen, Sigma-Aldrich, USA, 5 U/mL thrombin, Sigma-Aldrich, USA, 4.4 mM CaCl_2_, Sigma-Aldrich, USA, 0.4 mg/mL tranexamic-acid, Sigma-Aldrich, USA) at the density of 20 × 10^6^ cells/ mL corresponding to 2 × 10^6^ cells. The ECTs were generated by directly casting the cell-based fibrin gel on a PDMS membrane within the custom culture chambers (Supplemental Figure S2). ECTs were incubated 20 min at 37°C and 5% CO_2_ to allow complete gel polymerization and DMEM HG (Sigma-Aldrich, USA) supplemented with 10% FBS (Sigma-Aldrich, USA), 1% HEPES (Sigma-Aldrich, USA), 1% PS (Sigma-Aldrich, USA), 1% L-Glu (Sigma-Aldrich, USA) was added. From the second day onward, the culture media was replaced daily with DMEM LG (Sigma-Aldrich, USA) supplemented with 10% FBS (Sigma-Aldrich, USA), 1% HEPES (Sigma-Aldrich, USA), 1% PS (Sigma-Aldrich, USA) and 0.4 mg/mL tranexamic acid (Sigma-Aldrich, USA) to reduce fibrin degradation^52^ and remove waste products. After 3 days of static culture, ECTs were maintained for an additional 4 days either under static conditions (Control) or subjected to Mono and Asym ES modes. After a total of 7 days in culture (37°C, 95% humidity, 5% CO₂), the constructs were analyzed.

### Analyses

#### Immunofluorescence staining

Cells were washed with phosphate-buffered saline (PBS, Sigma-Aldrich, USA) and subsequently fixed with 4% paraformaldehyde (PFA, Sigma-Aldrich, USA) for 15 min. Immunofluorescence staining was then performed as follows. Cells were washed twice with PBS and permeabilized for 1 h at room temperature using a solution of 5% normal goat serum (Sigma-Aldrich, USA) and 0.25% Triton X-100 (Sigma-Aldrich, USA) in PBS. After two additional PBS washes, samples were incubated for 1 h in the dark with primary antibodies listed in the following paragraphs. Following primary antibody incubation, cells were washed with PBS and incubated with fluorescent secondary antibodies for 30 min in the dark. Nuclear staining was subsequently performed using DAPI (1:40 dilution, Invitrogen, Thermo Fisher Scientific, USA) for 15 min. All antibody incubations were carried out at room temperature in PBS containing 0.1% bovine serum albumin (BSA, Sigma-Aldrich, USA). Primary and secondary antibodies were used at a 1:200 dilution.

For each experimental condition, immunofluorescence images were acquired using a 40× objective Kinetix camera on a Nikon Crest V3 spinning-disk confocal microscope (Nikon, Japan).

##### Cell damage

Cell viability was assessed by labeling dead cells using Ethidium Homodimer-1 (EthD-1, Molecular Probes, Thermo Fisher Scientific, USA), a red fluorescent dye that exhibits strong fluorescence upon binding to nucleic acids. On day 7, prior to fixation, cells were incubated with 4 µM EthD-1 diluted in PBS for 40 min in the dark. Following incubation, the EthD-1 solution was removed, and cells were washed with PBS. Samples were then processed for subsequent immunofluorescence staining.

Apoptosis was evaluated by immunostaining for cleaved caspase-3 using a rabbit polyclonal IgG anti-cleaved caspase-3 antibody (1:200 dilution, Cleaved-Casp3, 9661S, Cell Signaling, USA), followed by an Alexa Fluor 488-conjugated anti-rabbit IgG secondary antibody (1:200 dilution, Life Technologies, Thermo Fisher Scientific, USA).

##### Cardiac maturation

Cardiac maturation was assessed using structural and functional markers. Samples were stained with mouse monoclonal IgG1 anti-sarcomeric α-actinin (1:200 dilution, Actn2, Abcam, ab9465, UK) to assess sarcomeric organization, and rabbit polyclonal IgG anti-Connexin-43 (1:200 dilution, Sigma-Aldrich, C6219, USA) to evaluate gap junction formation. Secondary antibodies included Alexa Fluor 546-conjugated anti-mouse IgG1 and Alexa Fluor 647-conjugated anti-rabbit IgG (1:200 dilution, Life Technologies, Thermo Fisher Scientific, USA).

#### Image analysis

##### Cell damage

To assess cell damage, the open-source digital pathology software QuPath (version 0.4.0) was used for image analysis. Total cell number was determined by counting DAPI positive nuclei. Cardiomyocytes and fibroblasts were identified by the presence or absence of Actn2, respectively. Cell viability was evaluated by identifying different stages and types of cell death based on marker expression. Cells were classified as follows: early apoptotic (Actn2^+^ or Actn2^−^, DAPI^+^, Cleaved-Casp3^+^ and EthD-1^−^), late apoptotic (Actn2^+^ or Actn2^−^, DAPI^+^, Cleaved-Casp3^+^ and EthD-1^+^), and non-apoptotic dead (Actn2^+^ or Actn2^−^, DAPI^+^, Cleaved-Casp3^−^ and EthD-1^+^). The cell populations were quantified and expressed as a percentage of total cardiomyocytes (Actn2^+^, DAPI^+^) and total fibroblasts (Actn2^−^, DAPI^+^) per image.

##### Cardiac maturation

Total cell count was determined by counting DAPI-positive nuclei using QuPath. Cardiomyocytes and cardiac fibroblasts (CFs) were identified as cells positive or negative for Actn2 or cardiac troponin-T (TnT), respectively.

Image analysis was performed using an open source software, Fiji software (version 2.16.0/1.54p).^53^ The “Intermodes” thresholding technique was used to segment Cx-43^+^ areas, while the “Li dark method” was applied to segment Actn2^+^ and cTnT^+^ regions. The percentage of Actn2^+^ and Cx-43^+^ areas were then normalized to the number of cardiomyocytes.^11,18,31,54–56^ Sarcomere length in cardiomyocytes was measured in Fiji by calculating the distance between two intensity peaks of adjacent sarcomeric filaments.^57–60^ Cardiomyocytes with organized sarcomeres and cardiomyocytes with aligned Cx-43 were manually counted with the Cell Counter tool of Fiji. Cardiomyocyte aspect ratios expressed as length-width ratio were manually measured with QuPath.

#### Troponin and lactate dehydrogenase release measurement

Cardiac cell damage was measured by quantifying the release of cardiac troponin I (cTnI), cTnT and lactate dehydrogenase (LDH) into the culture medium. Culture media were collected from each experimental condition on days 3, 5, and 7. After centrifugation at 1200 rpm, supernatants were collected and stored at −80°C until analysis. cTnT concentrations were measured using the high-sensitivity cTnT (hs-cTnT) assay on the Cobas pure e402 analyzer (Elecsys Troponin T hs Gen 5 STAT, Roche Diagnostics, Germany). Hs-cTnI levels were assessed with the ALINITY i-series analyzer (Alinity I STAT High Sensitive Troponin-I Reagent Kit, Abbott Diagnostics, Abbott Park, USA). LDH levels were measured on the Cobas pure c303 (Roche Diagnostics, Switzerland).

Reactive oxygen species (ROS) into the cell culture medium were quantified using the ROS-GLO H_2_O_2_ Assay (G8820, Promega, USA), following the manufacturer’s instructions. Luminescence was measured using a microplate luminometer (Synergy H1, Agilent Technologies, USA).

#### Cell viability assay

Cell viability was assessed using the MTT assay (Sigma, USA), which measures metabolic activity based on the ability of viable cells to reduce MTT (3-[4,5-dimethylthiazol-2-yl]-2,5-diphenyl tetrazolium bromide) into dark blue formazan crystals.^61^

NIH 3T3 fibroblasts^62^ were seeded in 48-well plates at a density of 20,000 cells/cm² and incubated at 37°C with 5% CO₂ for 48 h. Conditioned media were generated by subjecting LG DMEM (supplemented with 1% v/v HEPES buffer, 1% v/v penicillin/streptomycin, 1% v/v L-glutamine, and 1% v/v FBS) to ES (Monophasic, Symmetric Biphasic, Asymmetric Biphasic) or no stimulation for 48 h. These conditioned media were then applied to fibroblast cultures for 24 h. Fresh medium served as a negative control, while 0.1% ZDEC polyurethane film extract (RM-A, Hatano Research Institute, FDSC) was used as a positive cytotoxic control.

MTT dye (0.5 mg/mL, Sigma-Aldrich, USA) was diluted 1:10 in phenol red-free LG DMEM with the same supplements and added to each well for 4 h. The reaction was stopped using isopropanol containing 6 × 10⁻⁵M HCl, and absorbance was read at 575 nm using a microplate reader (Synergy H1, Agilent Technologies, USA). All conditions were tested in duplicate, and results are presented as the percentage of viable cells relative to the non-stimulated control.

#### Electrical functionality and cardiomyocyte contractility assessments

After 7 days of culture, the contractile activity of cardiomyocytes was evaluated in response to external electrical pacing by measuring two key electrical parameters: Excitation Threshold (ET) and Maximum Capture Rate (MCR).^31,63^ Pacing tests were conducted in a live-cell imaging microscope incubator (ZEISS X91, Olympus, Japan) at 37°C, 5% CO₂. Electrical pulses (1 Hz, 2 ms) were applied using the ELETTRA.^31^ Starting at 1 V/cm, voltage was gradually increased to identify the minimum electric field required for synchronized contraction. Once ET was established, 150% of this voltage was applied, and frequency was incrementally increased to identify the MCR, that is, the highest frequency the cells could follow without losing synchronicity.

Videos of paced cells were recorded at 30 frames per second (fps) using a 10× objective in the same live-imaging setup.

#### Functional inter-cellular communication

To evaluate the role of gap junction communication, functional parameters of cardiomyocytes were assessed before and after supplementing a pharmacological broad-spectrum gap junction inhibitor of connexins using carbenoxolone (Sigma-Aldrich). After 7 days of culture, baseline ET values were evaluated. Cells were treated with 100 µM carbenoxolone diluted in LG DMEM supplemented with 1% FBS, 1% penicillin/streptomycin, 1% L-glutamine, and 1% HEPES for 30 min at 37°C in 5% CO₂. Following treatment, ET measurements were repeated. The effect of gap junction inhibition was quantified by calculating the ratio of post- to pre-treatment values (ET₃₀_min_/ET₀).

#### Calcium transient

After assessing ET and MCR, intracellular calcium dynamics were evaluated using the Fluo-4 (Fluo-4 No-Wash Calcium Assay Kit, Invitrogen, Sigma-Aldrich, USA). Two chambers per condition, for 3 independent experiments, were selected for calcium imaging. Cells were washed with HBSS and incubated with 400 µL of Fluo-4 AM solution at 37 °C for 30 min. Following incubation, the dye was removed, and 2.5 mL Tyrode’s solution (140 mM NaCl, 5.4 mM KCl, 1 mM MgCl₂, 1.8 mM CaCl₂, 5.5 mM glucose, 5 mM HEPES in distilled water) was added for recording. Calcium transients were recorded during ES using a 40× objective Kinetix camera on a Nikon Crest V3 spinning-disk confocal microscope at 30 fps (Nikon, Japan; excitation at 488 nm, emission 500–550 nm) under live-cell conditions at 37°C. Fluorescence intensity over time was quantified using Fiji software.

Regions of interest (ROI) were drawn within contractile cardiomyocytes to measure calcium transient profiles. Fluorescence versus time plots were generated to assess intracellular calcium handling. Quantitative analysis included calculating the changes in fluorescence intensity (Δ*F*) between relaxed and contracted states. Moreover, peak delay (PD), defined as the maximum delay in fluorescence peakonset among different ROIs in response to a single pacing stimulus, was evaluated using a custom script (MATLAB).^31^

#### Contractility assessments

Cardiomyocyte contractile performance was evaluated by analyzing the live-imaging videos of electrically stimulated cells. Two key parameters were assessed: the peak amplitude (PA), indicating the maximum displacement of individual cardiomyocytes during contraction, and the contraction time delay (CTD), defined as the maximum delay in contraction onset across multiple cells in response to a single pacing stimulus. Cell displacement tracking was performed using the TrackMate plugin in Fiji (NIH, USA), and quantitative analysis was carried out using a custom script.^31^

For 3D ECTs, ET videos were analyzed using a custom open-source MATLAB-based graphical user interface (GUI), incorporating a Digital Image Correlation (DIC) algorithm.^64^ The DIC technique enables visualization and tracking of material deformation by generating a grid of reference points across the sample surface. Displacement and strain fields are calculated by correlating sequential video frames with a reference frame. Through the application of structural mechanics principles, this method enables precise quantification of local deformations and strain within the ECTs during contraction.

#### Proteomics

At the end of the 2D culture, cells were washed twice with PBS, collected and centrifugated for 5 min at 1200 rpm. Cells were then lysed in lysis buffer (1% sodium deoxycholate, 0.1M Tris, 10 mM TCEP, pH = 8.5) using ultra-sonication (10 cycles, Bioruptor, Diagenode, Belgium). Lysates were reduced for 10 min at 95°C and alkylated with 15 mM chloroacetamide for 30 min at 37°C. Proteins were digested by incubation with sequencing-grade modified trypsin (1/50, w/w; Promega, Madison, Wisconsin) for 12 h at 37°C. Tryptic digests were acidified (pH < 3) using TFA and cleaned up using iST cartridges (PreOmics, Germany) according to the manufacturer’s instructions. Samples were dried under vacuum and stored at −20°C until further use.

Dried peptides were resuspended in 0.1% aqueous formic acid and subjected to LC–MS/MS analysis using a Orbitrap Fusion Lumos Mass Spectrometer fitted with an EASY-nLC 1200 (both Thermo Fisher Scientific) and a custom-made column heater set to 60°C. Peptides were resolved using a RP-HPLC column (75 μm × 36 cm) packed in-house with C18 resin (ReproSil-Pur C18–AQ, 1.9 μm resin; Dr. Maisch GmbH, Germany) at a flow rate of 0.2 μL/min. The following gradient was used for peptide separation: from 5% B to 12% B over 5 min to 35% B over 90 min to 50% B over 25 min to 95% B over 2 min followed by 18 min at 95% B. Buffer A was 0.1% formic acid in water and buffer B was 80% acetonitrile, 0.1% formic acid in water.

The mass spectrometer was operated in DDA mode with a FAIMS Pro interface attached. FAIMS was run in standard resolution mode with 2 alternating CV voltages of −40 and −70 V. The total cycle time was 3 s (1.5 s per CV voltage) between master scans. Each master scan was acquired in the Orbitrap at a resolution of 120,000 FWHM (at 200 m/z) and a scan range from 375 to 1500 m/z followed by MS2 scans of the most intense precursors in the linear ion trap at “Rapid” scan rate with isolation width of the quadrupole set to 1.4 m/z. Maximum ion injection time was set to 50 ms (MS1) and 35 ms (MS2) with AGC target set to 1e6 and 1e4, respectively. Only peptide ions with charge state 2–5 were included in the analysis. Monoisotopic precursor selection (MIPS) was set to Peptide, and the Intensity Threshold was set to 5e3. Peptides were fragmented by HCD (Higher-energy collisional dissociation) with collision energy set to 35%, and one microscan was acquired for each spectrum. The dynamic exclusion duration was set to 30 s.

The generated raw files were converted to mzXML and split by the applied compensation voltage using the FAIMS MzXML Generator software package (PMID: 29969236). The mzXML -files were searched using MaxQuant (v1.6.17.0) against a Rattus norvegicus database (consisting of 31575 protein sequences downloaded from Uniprot on 20200417) spiked with the sequences of human Fibrinogen alpha, beta, and gamma chains (Uniprot accessions P02671, P02675, and P02679) and commonly observed contaminants using the following search criteria: full tryptic specificity was required (cleavage after lysine or arginine residues, unless followed by proline); 2 missed cleavages were allowed; carbamidomethylation (C) was set as fixed modification; oxidation (M); and acetylation (Protein N-term) were applied as variable modifications; mass tolerance of 20 ppm (precursor) and 0.5 Da (fragments); match between runs was enabled. The database search results were filtered to a false discovery rate (FDR) of 1% on the peptide and protein level. Quantitative analysis results from label-free quantification were processed using the SafeQuant R package v.2.3.2. (PMID: 27345528, https://github.com/eahrne/SafeQuant/) to obtain peptide relative abundances. This analysis included global data normalization by equalizing the total peak areas across all LC-MS runs, data imputation using the knn algorithm, summation of peak areas per protein and LC-MS/MS run, followed by calculation of protein abundance ratios. Only isoform specific peptide ion signals were considered for quantification. The summarized protein expression values were transformed from the linear to the log-scale and normalized by subtracting the respective columns medians differences to the grand median. Differential expression (DE) for proteins between conditions was obtained by applying empirical Bayes moderated t-Tests implemented in the R/Bioconductor limma package (PMID: 25605792). Considering the study design, all proteins showing p-value (adjusted by Benjamini-Hochberg method) <0.05 were considered DE. For pathway analysis, UniProt accession numbers were mapped to Human orthologs using Gene and Ortholog Location Finder (GOLF) function from RGD database (https://rgd.mcw.edu/rgdweb/ortholog/start.html) and pathway analysis was performed on MSigDb database (PMID: 16199517) using Competitive Gene Set Enrichment Analysis (Camera) in limma package. Pathways with FDR score lower than 0.1 were considered differentially regulated.

### Statistical analysis

All data are presented as mean ± standard deviation (SD). One-way and two-way ANOVA test was used for normally distributed populations (Figures 4(d)–(f), 5(c), (d), 6(e), 7(c), 8(a) and (b), S9A). For all other graphs, the non-parametric Mann–Whitney test or Kruskal-Wallis test was used for single and multiple comparisons, respectively. Statistical analyses were conducted using GraphPad Prism 10 (GraphPad Software, Inc., USA). Statistical significance was set at *p* < 0.05.

To compare the distribution of sarcomere lengths between experimental conditions, statistical analyses were performed in R (version 4.4.2) using packages (dplpr, tidyr, stringr, purrr, janitor). Inter-group comparisons of categorical proportions were assessed with Chi-square. Multiple comparisons were performed with Pairwise Fisher’s exact tests with Holm correction.

## Results

### Characterization of ES modes

Experimental measurements and simulated values of total delivered charge, residual voltage and total energy were compared across the three ES modes (Table 1). For each condition, the total delivered charge and total energy showed good agreement between measured and simulated values. The residual voltage was higher in absolute value for the experimental measurements, as the lump parameter model does not account for the non-ideal behavior of the components. The total delivered charge was the highest for the Mono ES mode, which also exhibits the most negative residual voltage (−790.9 ± 0.0 mV measured), indicating significant charge accumulation at the electrode double layer. In contrast, the Sym Bi ES mode delivered the lowest total charge (69.3 μC simulated, 59.8 ± 0.3 μC measured) and showed minimal residual voltage (89.8 ± 5.6 mV measured), refelecting its balanced nature. The Asym Bi ES mode delivered an intermediate charge (87.1 μC simulated, 89.7 ± 0.1 μC measured) with a moderate residual voltage (−193.6 ± 0.0 mV measured), showing a trade-off between charge delivery and reversibility.

**Table 1.** Electrical characterization of ES modes. Simulated and measured total delivered charge, residual voltage and total energy values for the three applied ES modes.

Electrical characterization	Simulation			Experimental measurement		
ES mode	Total delivered charge (μC)	Residual voltage (mV)	Total energy (μJ)	Total delivered charge (μC)	Residual voltage (mV)	Total energy (μJ)
Mono (+3 V/cm)	139.4	−278	415.2	133.1 ± 0.1	−790.9 ± 0.0	399.2 ± 0.2
Sym Bi (±1.5 V/cm)	69.3	37	103.7	59.8 ± 0.3	89.8 ± 5.6	89.1 ± 0.4
Asym Bi (+3/−1 V/cm)	87.1	−91	221.1	89.7 ± 0.1	−193.6 ± 0.0	226.8 ± 0.1

The total energy calculated from the measured voltage and current resulted the highest for Mono ES (415.2 μJ simulated, 399.2 ± 0.2 μJ measured) and the lowest for Sym Bi ES (103.7 μJ simulated, 89.7 ± 0.4 μJ measured). Asym Bi ES was characterized by an intermediate value of total energy (221.1 μJ simulated, 226.8 ± 0.1 μJ measured), notably lower than that of Mono ES. The experimental waveforms of current and voltage on the sensing resistors for the three ES modes are provided in Supplemental Figures S3 and S4.

### 2D cell culture experiments

#### ES modulated the molecular signature of cultured cells

**Figure 2.**
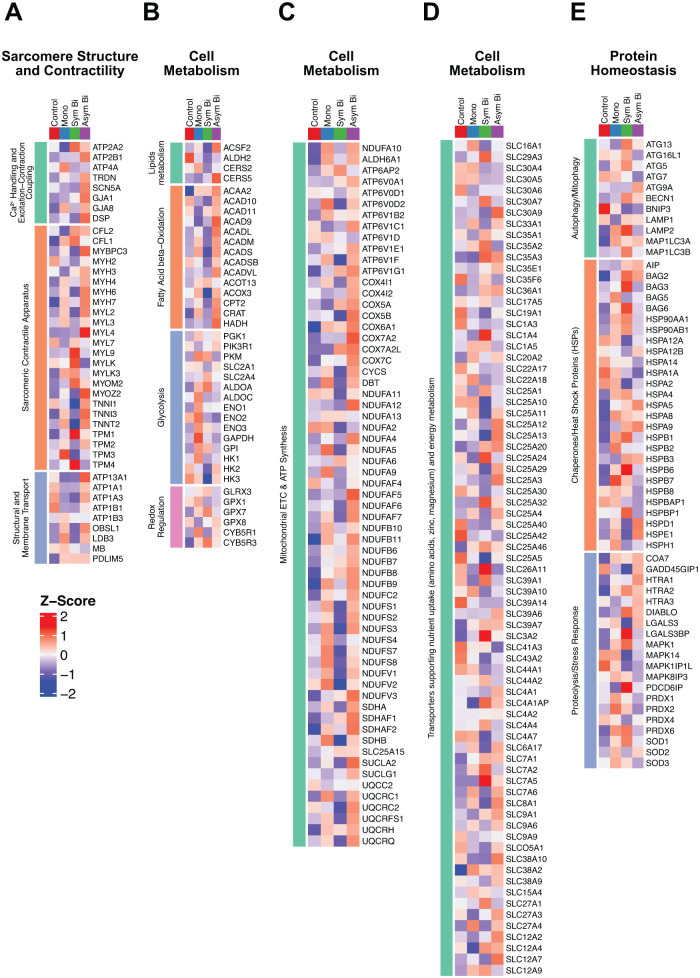
Effects of electrical stimulation on cardiac maturation, cell metabolism and homeostasis. Heatmaps of proteins related to sarcomere structure and contractility (a), cell metabolism (b–d), and homeostasis (e) across experimental groups: Control, Mono, Sym Bi, and Asym Bi. Protein selection was based on literature-curated lists relevant to engineered cardiac tissue. Each row represents a single protein; each column represents an individual group. Protein abundance values are defined as z-scores, calculated per protein across groups to highlight relative expression patterns; high expression is shown in red, low expression is shown in blue.

Mass spectrometry analysis of cardiac cells exposed to the three different ES (Mono, Sym Bi, and Asym Bi) modes and not electrically stimulated cells (Control) was performed. The 10% most variable proteins across the experimental groups are shown in Supplemental Figure S5. From the full set of proteins identified by mass spectrometry, we focused on a subset of known proteins typically expressed in engineered cardiac tissue (ECT),^23,24,65–69^ which we organized into four main functional categories based on their biological roles: (1) sarcomere structure and contractility; (2) cell death and survival; (3) protein related to cell homeostasis; and (4) cell metabolism. The relative level of these proteins was visualized as heat maps to highlight group-specific expression patterns (Figures 2 and S6).

No evident differences were observed between experimental groups in proteins related to cell death and survival (Supplemental Figure S6). In contrast, compared to the other experimental groups, the Asym Bi group exhibited the most pronounced changes in proteins associated with sarcomere structure and contractility, protein homeostasis, and cellular metabolism (Figure 2).

Specifically, the group Asym Bi showed high expression of proteins involved in Ca^2+^ handling and excitation-contraction coupling, as ATP2B1, ATP2B2, GJA1, and DSP, as well as components of the sarcomeric contractile apparatus including different myosin heavy and light chain proteins, and the cardiac troponins (TNNI3, TNNT2; Figure 2(a)). Interestingly, proteins involved in fatty acid β-oxidation were also more abundant in the Asym Bi group, which simultaneously exhibited lower levels of glycolytic proteins (Figure 2(b)), indicating a metabolic shift toward an oxidative phenotype, typical of more mature cardiomyocytes. In addition, the Asym Bi group displayed increased expression of proteins associated with the assembly, stability, and function of mitochondrial complexes IV, I, II, and III (e.g. COX6A1 COX7A2, COX7A2I, NDUFAF5, NDUFAF6, NDUFAF7, SDHAF1, SDHAF2, UQCRC2, UQCRQ) compared to the other conditions, further supporting enhanced bioenergetic capacity (Figure 2(c)). While the expression of transport proteins related to nutrient uptake and energy metabolism was mainly comparable in all groups, a slight increase was observed in the Asym Bi group (Figure 2(d)). Finally, proteins associated with autophagy/mitophagy, proteolysis/stress response, and chaperones were generally less abundant in the Asym Bi group, suggesting reduced cellular stress response activation comparing with the other conditions (Figure 2(e)).

##### Differential expression analysis

To further highlight the differences in protein abundance between experimental groups, pairwise DE analysis and Gene Ontology (GO) enrichment analysis for Biological Processes were performed.

##### Control versus stimulated conditions (Mono, Sym Bi, Asym Bi)

Compared to unstimulated controls, all ES conditions led to increased abundance of proteins involved in mitochondrial function and, to varying degrees, structural maturation. Specifically, Mono stimulation compared to Control showed increased expression of mitochondrial complex components, such as COX6.1, NDUFB9, and NDUFB11 (Supplemental Figure S7A), with GO: BP enrichment for pathways related to oxidative phosphorylation, ATP biosynthesis, and sarcomere organization (Supplemental Figure S7B).

In addition to the upregulation of mitochondrial proteins, Sym Bi versus Control also showed an increased expression of sarcomere structural proteins as MYH11, ACTA2, and tropomyosin 1 and 4 (TPM1, TPM4), as well proteins involved in the maintenance of protein homeostasis (BAG2, HSPB1, HSPB6, HTRA2, and PRDX6; Supplemental Figure S7C). However, GO: BP analysis showed an enrichment limited to cytoplasmic translation pathways (Supplemental Figure S7D).

**Figure 3.**
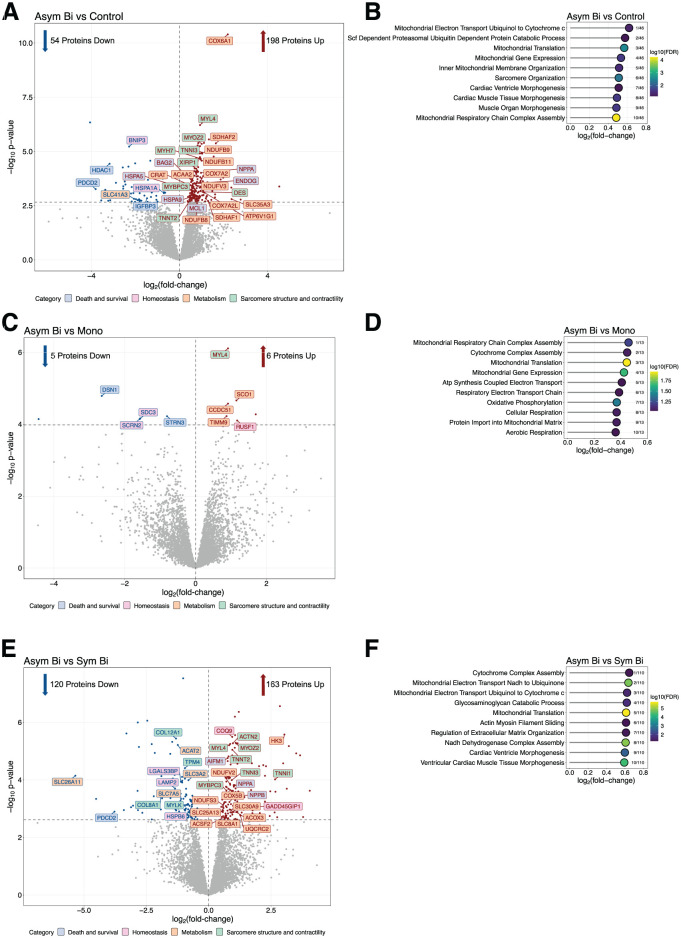
Pairwise comparisons of differentially expressed proteins and GO: BP enrichment analyses. Pairwise comparisons of Asym Bi versus Control (a and b), Asym Bi versus Mono (c and d), and Asym Bi versus Sym Bi (c and d) are shown. In the volcano plots (a, c, and e), the x-axis represents log_2_ (fold change) and the y-axis represents −log_10_ (p-value) for the differential expression analysis. In the upper right corner, the number of proteins increased and in the upper left corner, the number of proteins decreased. Significance was determined based on the adjusted p-value (FDR) < 0.05 and shown as a vertical gray dotted line. Red and blue dots indicate proteins with significantly increased or decreased abundance, respectively, while gray dots represent proteins without significant changes. Protein names outlined in red or blue denote significant increases or decreases in protein levels, respectively. Proteins are grouped into four categories based on their function: blue (death and survival), pink (homeostasis), orange (metabolism), and green (sarcomere structure and contractility). GO:BP enrichment analysis results (b, d, and f) are plotted with the x-axis indicating log_2_ (fold change). Pathways with a false discovery rate (FDR) below 0.1 were considered significantly enriched.

In the DE analysis between Asym Bi and Control, Asym Bi stimulation resulted in elevated expression of mitochondrial proteins associated with fatty acid oxidation and oxidative phosphorylation. These included subunits of cytochrome c oxidase (complex IV: COX6A1, COX7A2, COX7A2L), NADH-dehydrogenase (complex I: NDUFB8, NDUFB9, NDUFB11, NDUFV), and of succinate dehydrogenase (complex II: SDHAF1, SDHAF2, and SLC35A3; Figure 3(a)). GO: BP analysis further confirmed enrichment in pathways related to mitochondrial electron transport, respiratory chain complex assembly, and mitochondrial translation (Figure 3(b)). Additionally, several sarcomeric proteins, such as MYBPC3, MYH7, MYL4, MYOZ2, TNNI3, and TNNT2, were significantly upregulated in the Asym Bi group, with corresponding enrichment in biological processes such as sarcomere organization and cardiac muscle morphogenesis (Figure 3(a) and (b)).

##### Sym Bi versus mono

In the Sym Bi versus Mono comparison, Sym Bi stimulation resulted in increased expression of mitochondrial respiratory complexes (NDUFS3, NDUFA5, SDHB) along with solute carrier proteins involved in β-oxidation (SLC-proteins; Supplemental Figure S7E). GO:BP analysis corroborated these results, showing enrichment in pathways related to electron transport, ATP synthesis, and overall metabolic activity (Supplemental Figure S7F). Changes in levels of sarcomeric organization and functional proteins were less evident: while tropomyosin isoforms (TPM1, TPM4) and MYH11 were significantly higher, core of sarcomeric proteins such as TNNC1 and TNNT2 were lower in the Sym Bi group compared to the Mono group (Supplemental Figure S7E). Additionally, proteins involved in extra cellular matrix (ECM) organization as COL1A1 and COL12A1, were elevated in the Sym Bi compared to the Mono. GO:BP analysis confirmed enrichment in ECM assembly and collagen fibril organization (Supplemental Figure S7F).

##### Asym Bi versus mono

Although differential expression analysis between Asym Bi and Mono groups revealed only a limited number of significantly regulated proteins (Figure 3(c)), GO:BP enrichment demonstrated robust activation of mitochondrial metabolic pathways, including mitochondrial translation, respiratory chain complex assembly, mitochondrial gene expression, and oxidative phosphorylation (Figure 3(d)).

##### Asym Bi versus sym Bi

Compared to Sym Bi, Asym Bi stimulation resulted in greater upregulation of mitochondrial and fatty acid metabolism-related proteins (ACSF2, ACOX3, COX5B, NDUFS3, NDUFV2; Figure 3(e)). Consistent with this, GO: BP analysis showed enrichment for mitochondrial translation, electron transport, and NADH dehydrogenase complex assembly pathways (Figure 3(f)). Sarcomeric proteins, such as MYBPC3, MYL4, MYOZ2, TNNI3, and TNNT2, were significantly more abundant in Asym Bi group, while MYLK and TPM4 were reduced (Figure 3(e)). GO analysis further revealed enrichment in pathways related to actin-myosin filament sliding, cardiac morphogenesis, and ECM remodeling (Figure 3(f)). Changes in proteins related to homeostasis were modest, with decreased levels of LAMP2 and HSBP6 and increased levels of PDCD2 in Asym Bi condition compared to Sym Bi (Figure 3(e)).

#### ES preserved cell integrity and viability

**Figure 4.**
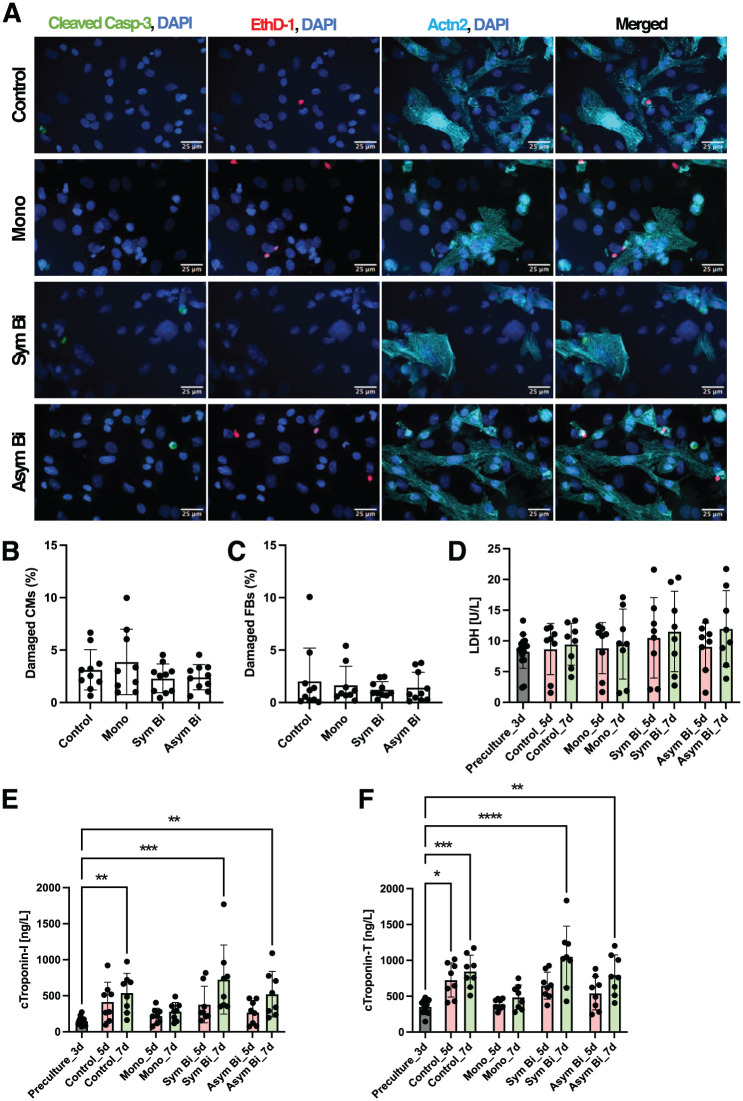
Effects of electrical stimulation on cell viability and damage analysis. (a) Immunofluorescence images of damaged cardiac cells for the different culture conditions. The images show cleaved Caspase-3 (Cleaved-Casp3, green), Ethidium Homodimer-1 (EthD-1, red) in separated images and the merged signals with sarcomeric α-actinin (Actn2, cyan) and the nuclei stained with DAPI (blue) for each experimental group. Scale bar = 25 μm. (b) Cardiomyocyte damage analysis and (c) Fibroblast damage analysis defined by the sum of early apoptotic cells identified as Cleaved-Casp3^+^ and EthD-1^−^ (Control = 8 replicate, Mono = 7 replicates, Sym Bi = 8 replicated, Asym Bi = 8 replicates), apoptotic dead cells as Cleaved-Casp3^+^ and EthD-1^+^ (Control = 8 replicate, Mono = 7 replicates, Sym Bi = 8 replicated, Asym Bi = 8 replicates) and non-apoptotic dead cells as Cleaved-Casp3^−^ and EthD-1^+^ (Control = 10 replicate, Mono = 9 replicates, Sym Bi = 10 replicated, Asym Bi = 10 replicates). Cardiomyocytes were identified as Actn2^+^ and DAPI^+^ and fibroblasts as Actn2^−^ and DAPI^+^. (d) Quantification of LDH release at the end of the preculture (*n* = 16 replicates) and cultured for 5 and 7 days under Control (*n* = 8 replicates), Mono (*n* = 8 replicates), Sym Bi (*n* = 8 replicates) and Asym Bi (*n* = 8 replicates). (e and f) Quantification of cTnI and cTnT release of cells at the end of the preculture (*n* = 16 replicates) and cultured for 5 and 7 days under Control (*n* = 8 replicates), Mono (*n* = 8 replicates), Sym Bi (*n* = 8 replicates) and Asym Bi (*n* = 8 replicates). Statistical analysis was performed using (b and c) nonparametric Kruskal-Wallis test and (d–f) a two-way ANOVA test. Asterisks (*) denote statistical significance (*p < 0.05, **p < 0.005, ***p < 0.0005, ****p < 0.0001).

Damaged cells were quantified as the total percentage of early apoptotic (EthD-1^−^, Cleaved-Casp3^+^, DAPI^+^), apoptotic dead (EthD-1^+^, Cleaved-Casp3^+^, DAPI^+^) and sudden dead (EthD-1^+^, Cleaved-Casp3^-^, DAPI^+^) cardiomyocytes (Actn2^+^; Figure 4(a) and (b) and S4) or fibroblasts (Actn2^-^; Figure 4(c) and S4). Across all three ES waveforms, cell damage was minimal and comparable to that of unstimulated controls, indicating that the ES protocols did not induce apoptosis-related cell damage or compromise overall cell viability.

Cell damage was also investigated by measuring LDH and cardiac troponins (cTnI and cTnT) levels in the cell supernatant. As shown in Figure 4(d), LDH, levels remained comparable across all experiment groups at each time point: after 3 days of preculture, after 2 days of ES (day 5), and after an additional 2 days of stimulation (day 7).

In the Sym Bi and Asym Bi ES conditions, the release of both cTnI (Figure 4(e)) and cTnT (Figure 4(f)) showed a time-dependent increase; however, differences were not statistically significant. At days 5 and 7, cTnI and cTnT levels were comparable among the Control, Sym Bi, and Asym Bi groups, while the Mono group displayed lower biomarker levels, albeit not significantly different. Comparing preculture values of cTnI (354.63 ± 92.61 ng/L) and cTnT (147.16 ± 51.61 ng/L) with end-point values, significant increases were observed in the Control (cTnI: 845.63 ± 227.40 ng/L, cTnT: 539.66 ± 271.24 ng/L), Sym Bi (cTnI: 1052.88 ± 425.12 ng/L, cTnT: 725.04 ± 479.21 ng/L) and Asym Bi (cTnI: 796.00 ± 279.52 ng/L, cTnT: 521.74 ± 316.02 ng/L) groups. In contrast, the Mono group showed the lowest levels at the end of the experiment (cTnI 486.13 ± 170.34 ng/L, cTnT (281.22 ± 124.47 ng/L). Although not statistically significant, cTnT consistently exceeded cTnI across all groups, yielding a cTnI/cTnT ratio of approximately 0.5 (Supplemental Figure S8), indicating an absence of necrotic cell death.^70^

Given that ES can generate potentially toxic by-products such as ROS via non-reversible faradaic reactions at the electrodes, ROS production was analyzed under different stimulation conditions. Bioluminescence assay showed no significant differencecs in ROS levels across all ES groups, including the negative control (Supplemental Figure S8).

To further confirm that ES had no detrimental effect on cell viability, an MTT assay was performed on human fibroblasts cultured in conditioned media previously exposed to the different ES modes. Fibroblast viability remained comparable to that of cells treated with non-stimulated conditioned medium (Supplemental Figure S8).

#### Asymmetric biphasic ES enhanced cardiac maturation

**Figure 5.**
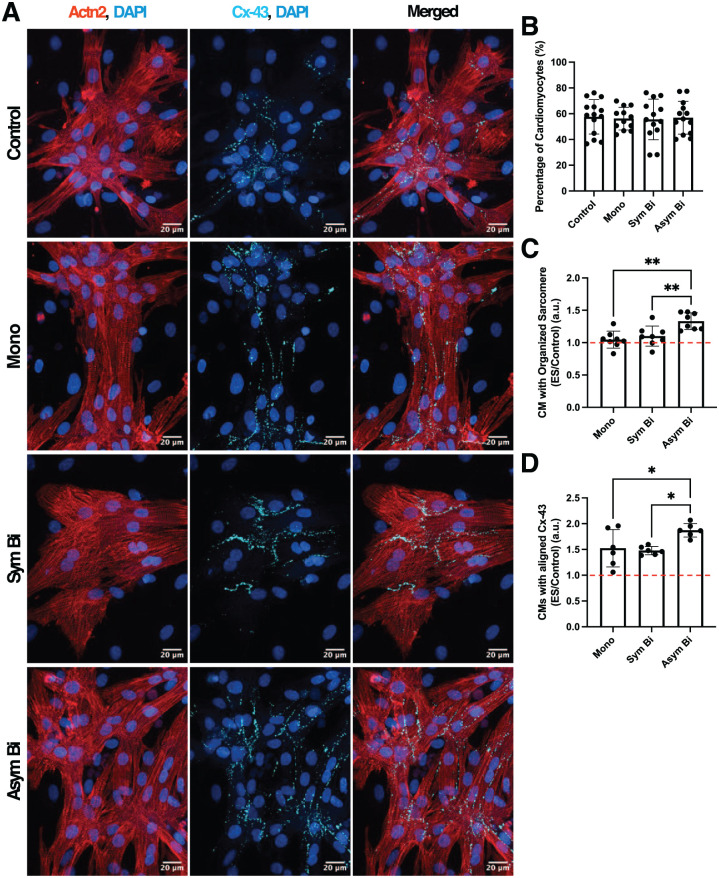
Effects of electrical stimulation on cardiac maturation. (a) Immunofluorescence images of cardiac cells for the different culture conditions. The images show the Connexin-43 (Cx-43, cyan), Sarcomeric α-actinin (Actn2, red) in separated images and the merged signals with nuclei stained with DAPI (blue) for each experimental group. Scale bar = 20 μm. (b) percentage of cardiomyocytes at the end of the culture for Control (*n* = 14 replicates); Mono (*n* = 12 replicates), Sym Bi (*n* = 13 replicates) and Asym Bi (*n* = 13 replicates). (c) Fold increase of the percentage of cardiomyocytes characterized by an organized sarcomere for the electrically stimulated groups Mono (n = 8 replicates), Sym Bi (*n* = 8 replicates) and Asym Bi (*n* = 8 replicates). (d) Fold increase of the percentage of cardiomyocytes characterized by the presence of aligned Cx-43 for the electrically stimulated groups Mono (*n* = 6 replicates), Sym Bi (*n* = 6 replicates) and Asym Bi (n = 6 replicates). Statistical analysis was performed using (b) nonparametric Kruskal-Wallis test, (c) one-way ANOVA test and (d) one-way ANOVA test. Asterisks (*) denote statistical significance (*p < 0.05, **p < 0.01).

To evaluate cardiac maturation, the presence of key cardiac proteins, namely the gap-junction protein Cx-43 and the contractile protein Actn2 was investigated (Figure 5(a)). Quantification of Actn2^+^ cells revealed that the proportion of cardiomyocytes was comparable across all experiment groups, averaging around 60% (Figure 5(b)). Although, sarcomere length (Supplemental Figure S9A) and the Actn2^+^ area normalized to the number of cardiomyocytes (Supplemental Figure S9C) were similar across all groups, indicating equivalent cardiomyocyte density and sarcomeric content, Asym Bi significantly increased the percentage of cardiomyocytes with organized sarcomere compared to the other experimental groups (Figure 5(c)). Additionally, even if the other electrically stimulated groups, Mono and Sym Bi, exhibited more organized sarcomeric structures compared to the Control group, indicating enhanced structural maturation induced by ES (Figure 5(c)), there were no differences between them. Moreover, by analyzing the distribution of sarcomere lengths across defined ranges, it was observed that the Asym Bi group was characterized by a smaller proportion of cardiomyocytes with short sarcomeres (1.1–1.6 µm), characteristic of less mature cells, and a higher proportion of Cardiomyocytes with longer sarcomeres within the 1.6–2.0 µm and 2.0–2.5 µm ranges, indicative of greater structural maturation (Supplemental Figure S9B).

Cx-43 expression, normalized to cardiomyocyte number and presented as fold change relative to the Control, was elevated (>1) in all electrically stimulated conditions (Supplemental Figure S9D), with the Mono and Asym Bi groups showing the highest levels. Immunofluorescence imaging revealed that Cx-43 in the ES groups was predominantly aligned as opposed to the diffuse, peri-nuclear distribution observed in the Control group. In particular, Asym Bi significantly increase the percentage of cardiomyocytes characterized by an aligned Cx-43 compared to the other experimental groups (Figure 5(d)).

#### Asymmetric biphasic ES enhanced cardiac electrical functionality and contractility

**Figure 6.**
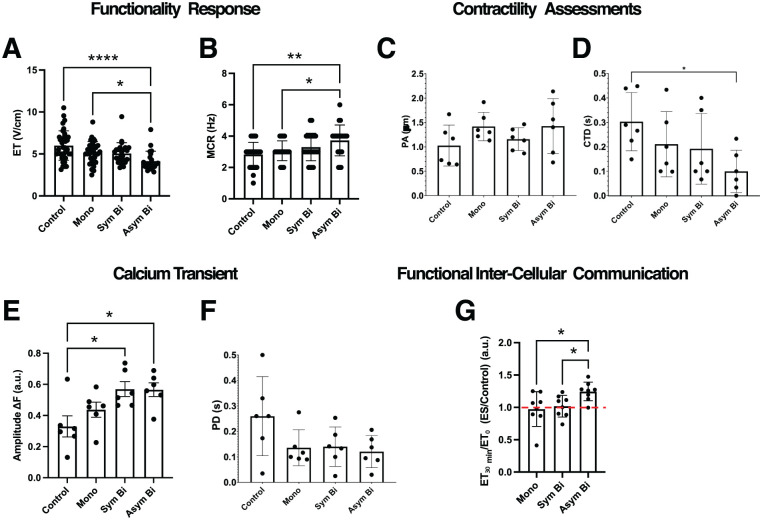
Effects of ES on 2D cardiac cell functionality and contractility. (a) Excitation threshold (ET) and (b) Maximum capture rate (MCR) of cardiomyocytes for the different culture conditions: Control (*n* = 40 replicates), Mono (*n* = 31 replicates), Sym Bi (*n* = 24 replicates) and Asym Bi (*n* = 22 replicates). Results from 12 independent experiments. Cardiomyocyte contractility analysis of (c) Peak amplitude (PA) and (d) contraction time delay (CTD) for the different culture conditions: Control (*n* = 6 replicates), Mono (*n* = 6 replicates), Sym Bi (*n* = 6 replicates) and Asym Bi (*n* = 6 replicates). (e) Effects of ES on calcium transient. Control (*n* = 6 replicates), Mono (*n* = 6 replicates), Sym Bi (*n* = 6 replicates) and Asym Bi (*n* = 6 replicates). (f) Calcium Peak Delay (PD) of calcium transient signals following a single pacing pulse of different cardiomyocyte groups: Control (*n* = 6 replicates), Mono (*n* = 6 replicates), Sym Bi (n = 6 replicates) and Asym Bi (n = 6 replicates). (g) Effects of ES on gap junction communication. Data expressed as fold increase with the unstimulated cells. Control (n = 8 replicates), Mono (*n* = 8 replicates), Sym Bi (*n* = 8 replicates) and Asym Bi (n = 8 replicates). Statistical analysis was performed using A) nonparametric Kruskal-Wallis test, (b) nonparametric Kruskal-Wallis test, (c) nonparametric Kruskal-Wallis test, D) nonparametric Kruskal-Wallis test, (e) one-way ANOVA test, (f) nonparametric Kruskal-Wallis test, (g) unpaired t-test. Asterisks (*) denote statistical significance (*p < 0.05, **p < 0.01, ****p < 0.0001).

Following 7 days of culture under either Control or ES conditions, electrical functionality was assessed by evaluating cell response to external electrical pacing. Cardiac cells cultured under Asym Bi ES exhibited synchronous contractions at a significantly lower ET (4.15 ± 1.17 V/cm) compared to the Control (6.01 ± 1.75 V/cm) and the Mono (5.13 ± 1.33 V/cm) groups (Figure 6(a); Supplemental Videos S5–S8). Regarding the MCR, all ES groups showed an increasing trend relative to the Control (2.83 ± 0.77 Hz), and only the Asym Bi group achieved a significantly higher MCR (3.73 ± 0.98 Hz) compared to both the Control and Mono groups (3.06 ± 0.63 Hz). Although MCR in the Asym Bi group was slightly higher than in the Sym Bi group (3.30 ± 0.88 Hz), the difference was not statistically significant, with all ES groups reaching approximately 3 Hz (Figure 6(b)).

Contractility performance was evaluated using the peak amplitude (PA) and the contraction time delay (CTD) from videos recorded during electrical pacing (Supplemental Figure S10). PA was elevated in all ES groups compared to the Control (1.026 ± 0.420 µm), suggesting a positive effect of ES, although these differences were not statistically significant (Figure 6(c)). Specifically, Mono ES (1.418 ± 0.291 µm) and Asym Bi ES (1.426 ± 0.564 µm) stimulated cells yielded greater PA than the Sym Bi group (1.158 ± 0.234 µm). For contraction synchronization, the Asym Bi group showed a significantly lower CTD (0.099 ± 0.087 s) compared to the Control (0.303 ± 0.119 s). All ES groups showed lower CTD compared to Control with both Mono (0.211 ± 0.133 s) and Sym Bi (0.192 ± 0.144 s) groups reporting similar values, overall indicating more synchronized contractions under all ES groups (Figure 6(d)).

To evaluate the effect of ES on excitation-contraction coupling, intracellular calcium dynamics were measured using the calcium-sensitive dye Fluo-4 (Figure 6(e)). The fluorescence amplitude (Δ*F*), defined as difference between relaxed and contracted states, was significantly higher in the Asym Bi (0.57 ± 0.11 a.u., Supplemental Video S4) and Sym Bi (0.57 ± 0.12 a.u., Supplemental Video S3) groups compared to Control (0.33 ± 0.17 a.u., Supplemental Video S1). Although ΔF in the Mono group (0.44 ± 0.12 a.u., Supplemental Video S2) was lower than in the biphasic groups, the differences were not statistically significant. Peak delay (PD) analysis of calcium transients showed reduced PD in all ES groups (Mono: 0.136 ± 0.071 s, Sym Bi: 0.140 ± 0.077 s, and Asym Bi: 0.121 ± 0.063 s) compared to the Control (0.260 ± 0.155 s), though without significant differences among the ES groups (Figure 6(f)). These findings suggest that ES enhances calcium handling and improves excitation-contraction coupling, regardless of waveform.

Finally, to investigate the role of gap junction-mediated communication, connexins were inhibited with carbenoxolone. Following connexins blockade, cells in the Asym Bi group exhibited a significant increase in ET compared to their pre-treatment values, indicating substantial impaired electrical responsiveness. In contrast, changes in ET in the Mono and Sym Bi groups were comparable to those observed in the Control (Figure 6(g)).

### 3D cell culture experiments

2D cardiac cell cultures have significant limitations, particularly their inability to replicate the native myocardium’s 3D architecture and cell–extracellular matrix interactions. To address these shortcomings, 3D culture systems using fibrin-based hydrogels have been developed.

Given the minimal differences observed between the Mono and Sym Bi stimulation groups in 2D experiments, in terms of cardiac maturation (sarcomere length, percentage of cardiomyocytes with organized sarcomere, percentage of cardiomyocytes with aligned Cx-43 and proteomics) and functionality (ET, MCR, calcium handling and the effects on gap junction communication), subsequent 3D experiments focused on comparing Mono and Asym Bi ES modes to assess potential differences in cardiac tissue performance between the two waveforms.

#### Asymmetric biphasic ES enhanced ECT cardiac maturation

**Figure 7.**
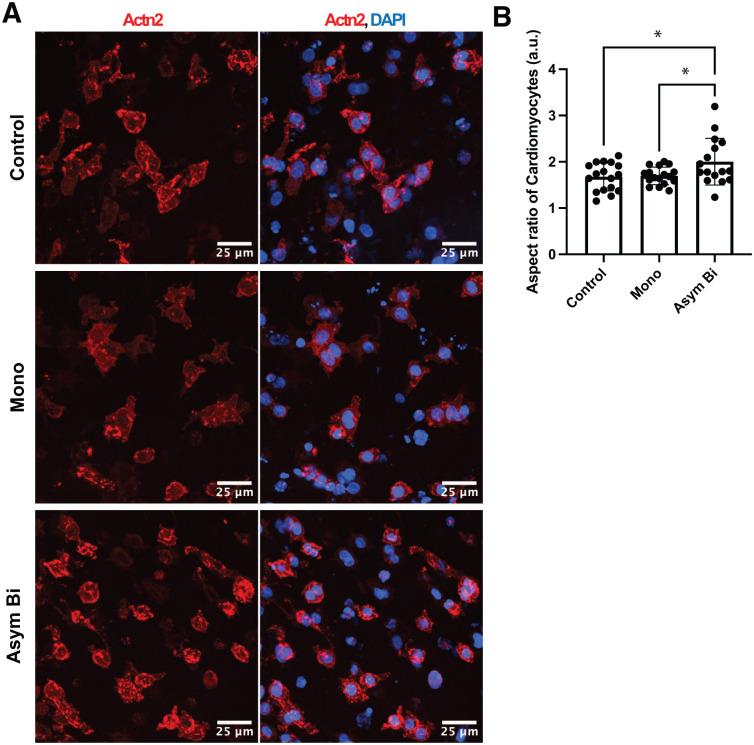
Effects of ES on ECTs maturation. (a) Representative immunofluorescence images of cardiac cells in ECTs cultured with Mono, Asym Bi or no stimulation. The cells were stained for sarcomeric α-actinin (Actn2, red) while the nuclei were stained with DAPI (blue). Scale bar = 25 μm. (b) Cardiomyocyte aspect ratio for the different culture conditions: Control (*n* = 3 replicates), Mono (*n* = 3 replicates) and Asym Bi (n = 3 replicates). Statistical analysis was performed using (b) Mann-Whitney test and (c) one-way ANOVA test. Asterisks (*) denote statistical significance (*p < 0.05).

To assess the maturation of electrically stimulated and non-stimulated ECTs (Supplemental Figure S2), immunofluorescence staining was performed for the cardiac-specific protein Actn2 (Figure 7(a)). Quantification of Actn2^+^ cells revealed that the proportion of cardiomyocytes was comparable across all experiment groups (Control: 53.9 ± 3.6%, Mono: 60.7 ± 3.1%, and Asym Bi: 59.2 ± 1.4%). The Asym Bi group exhibited a significantly larger Actn2^+^ area compared to the Mono group (Figure 7(b)). Although cardiomyocytes displayed predominantly rounded morphologies across all conditions, Asym Bi significantly improved the cardiomyocyte elongation (Figure 7(b)).

#### Asymmetric biphasic ES enhanced ECT electrical functionality and contractility

**Figure 8.**
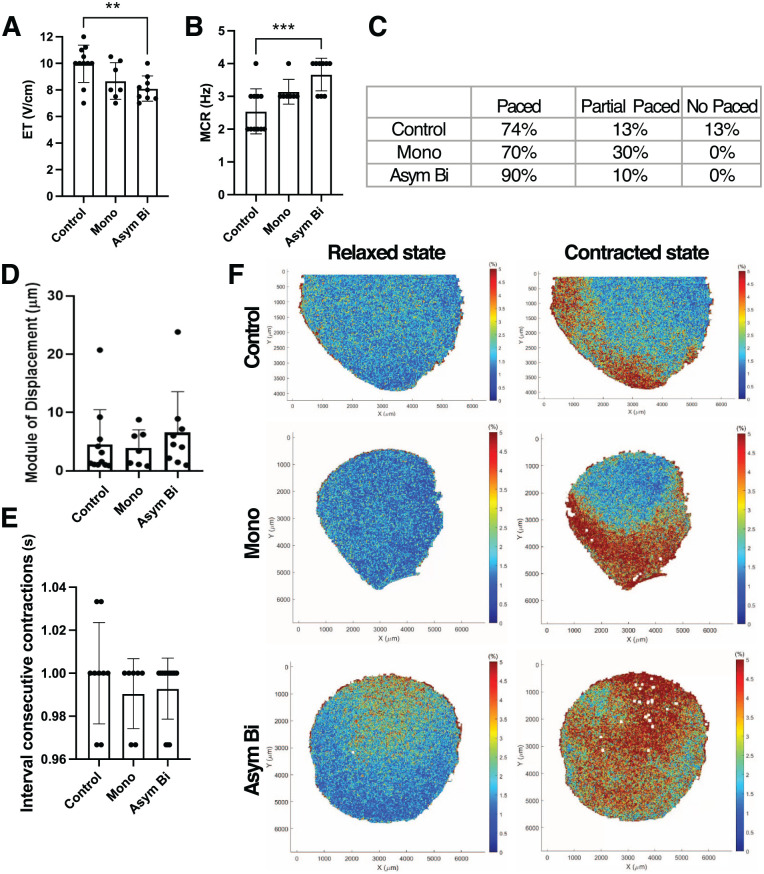
Effects of ES on ECTs functionality and contractility. (a) Excitation threshold (ET) and (b) Maximum capture rate (MCR) in different culture conditions: Control (*n* = 11 replicates), Mono (*n* = 7 replicates) and Asym Bi (n = 8 replicates). (c) Table distinguishing the whole (paced), the partial or the no response of ECTs following an external electrical stimulation for Control (*n* = 15 replicates), Mono (*n* = 10 replicates) and Asym Bi (*n* = 10 replicates). (d) Module of displacement of ECTs for the three experimental groups. (e) Time interval between consecutive contraction of the three experimental groups. (f) ECT strain heat maps in the three experimental groups under relaxed and contracted state. Statistical analysis was performed using A) and (b) one-way ANOVA test, (d) nonparametric Kruskal-Wallis test, (e) nonparametric Kruskal-Wallis test. Asterisks (*) denote statistical significance (**p < 0.01, ***p < 0.001).

ECTs cultured with Asym Bi stimulation exhibited lower ET values (8.11 ± 0.95 V/cm) compared to Mono stimulation (8.67 ± 1.38 V/cm), and significantly lower than the Control group (9.97 ± 1.40 V/cm; Figure 8(a)). Additionally, the Asym Bi group demonstrated superior MCR (3.67 ± 0.50 Hz) compared to the Mono (3.14 ± 0.38 Hz) and Control (2.55 ± 0.69 Hz) groups (Figure 8(b)). Under external pacing, 90% of Asym Bi-stimulated ECTs contracted synchronously, a marked improvement over the Mono (70%) and Control (73%) groups (Figure 8(c)).

Regarding construct displacement under ES, no statistically significant differences were observed among the experimental groups (Figure 8(d)). However, the Asym Bi group displayed a trend toward increased displacement (6.56 ± 7.00 μm) compared to the Mono (3.94 ± 3.09 μm) and Control (4.51 ± 5.97 μm) groups. Moreover, high displacement events were observed only in the Asym Bi (23.82 μm) and Control (20.72 μm) groups.

Beating synchrony was further evaluated by analyzing videos processed via DIC to measure the time interval between two consecutive peaks in average strain. All samples followed the stimulation frequency of 1 Hz (Figure 8(e)) with coefficients of variation in peak intervals of 2.4% for the Control, and 1.6% and 1.4% for the Mono and Asym Bi groups, respectively. Finally, strain distribution during contraction was assessed. Control ECTs showed deformation predominantly at the construct edges, whereas both stimulated conditions displayed central contraction (Supplemental Videos S9–S11). Notably, the Asym Bi ECTs exhibited a more uniform strain pattern, suggesting enhanced functionality (Figure 8(f)), which was 3.4 times higher compared to the Control. On the other hand, Mono ECTs exhibited an increase of strain of 2.1 times compared to the Control.

## Discussion and conclusion

ES has long been explored as a cue to promote the functional maturation of cardiomyocytes *in vitro*. However, achieving adult-like phenotypes remains a significant challenge. Studies have consistently demonstrated that applying Mono or Sym Bi ES *in vitro* can influence the rate, duration, and number of action potentials, improve sarcomere organization, and establish functional gap junctions. These changes promote cell–cell coupling and calcium handling, thereby, increasing the electrical and contractile functionality of stimulated cells.^31,45,71,72^

In this study, we introduced a novel asymmetric biphasic waveform designed to combine the advantages of Mono and Sym Bi stimulations. The positive half-wave mimics Mono stimulation, inducing cell depolarization, while the smaller negative phase, similar to Sym Bi, partially discharges the electrode and helps limit the accumulation of faradaic by-products. Lumped-parameter modeling and empirical measurements confirmed that Asym Bi delivered intermediate total charge (89.7 ± 0.1 μC measured) and moderate residual voltage (−193.6 ± 0.0 mV ), contrasting with the high charge (133.1 ± 0.1 μC) and large negative residual voltage (−790.9 ± 0.0 mV) of Mono ES, and the minimal residual voltage (89.8 ± 5.6 mV) but halved charge (59.8 ± 0.3 μC) of Sym Bi ES (Table 1). Moreover, Asym Bi ES delivered an intermediate total energy (226.8 ± 0.1 μJ), notably lower than the total energy of Mono ES (399.2 ± 0.2 μJ), but more than double than that of Sym Bi ES (89.7 ± 0.4 μJ).

We investigated the effects of these ES waveforms on 2D cardiac monolayers in terms of cell damage, cardiac maturation, functionality, and metabolism. To our knowledge, this is the first direct comparison of an Asym Bi waveform against conventional Mono and Sym Bi waveforms.

Notably, none of the ES conditions, Mono, Sym Bi or Asym Bi, induced significant cellular damage. Apoptosis and necrosis markers (EthD-1, Cleaved-Casp3) as well as LDH levels, cTnI and cTnT in the medium, were comparable across all groups. ROS levels were also similar, likely due to frequent medium exchange (every 48 h), preventing by-product accumulation. In addition, the use of carbon rod electrodes, known for their high biocompatibility, charge transfer and resistance to corrosion^38,40^ may have further minimized toxic effects. MTT cytotoxicity assay and proteomic analysis further supported these findings, with stress- and apoptosis-related proteins unchanged in the stimulated groups, particularly under Asym Bi, confirming that the ES waveforms applied didn’t have harmful effects on cardiac cells.

While cardiomyocyte number and other cardiac maturation parameters, such as sarcomere length and Actn2^+^ area, were similar among groups, Cx-43 levels were significantly higher in electrically stimulated cells, with Cx-43 predominantly aligned along the cell membranes. In contrast, non-stimulated cells showed more cytoplasmic Cx-43 distribution, indicative of immature or non-functional gap junctions. Importantly, Asym Bi exhibited a significantly higher percentage of cardiomyocytes with organized sarcomeres and a higher percentage of cardiomyocytes showing aligned Cx-43 compared to all other experimental conditions, highlighting the superior structural organization and intercellular coupling achieved under Asym Bi stimulation. These observations align with previous studies reporting that ES promotes Cx-43 expression and its membrane localization, thus enhancing intercellular electrical connectivity.^23,29–31^ The enhanced maturation of electrically stimulated cardiomyocytes was further supported at the proteomic level. In particular, Asym Bi exhibited the highest presence of proteins involved in calcium handling and excitation–contraction coupling, including TRDN, GJA1, GJA8 and DSP. Asym Bi also showed highest levels of sarcomeric contractile proteins such as TNNI3, TNNT2, MYL2, and MYH6, as well as structural and membrane transport proteins like ATP13A1 (Figure 3(f)).

Mass spectrometry analysis revealed a metabolic shift toward fatty acid oxidation in the Asym Bi group, a characteristic property of more mature cardiomyocytes. In contrast, cells in the Control, Mono and Sym Bi groups continued to rely more on glycolysis, typical of immature metabolic states. Moreover, Asym Bi stimulation led to increased expression of proteins involved in calcium handling and mitochondrial ATP synthesis, further indicating enhanced functional competence and bioenergetic efficiency.

The electrical functionality tests revealed that cardiomyocytes cultured under Asym Bi stimulation had the lowest ET values, significantly different from those in the Control and Mono groups (Figure 6(a)). Additionally, the MCR was significantly higher in the Asym Bi group (Figure 6(b)), reflecting improved electrical responsiveness and pacing reproducibility. In contrast, Mono and Sym Bi groups showed similar performances.

Contractility analysis confirmed the positive functional effects of ES. In particular, Asym Bi stimulation exhibited higher PA and shorter CTD (Figure 6(c) and (d)), indicating stronger and more synchronized contractions. Fluo-4 calcium imaging analysis confirmed increased calcium transient amplitudes and reduced peak delays in stimulated groups, with Sym Bi and Asym Bi groups showing a trend toward superior calcium handling. However, the Asym Bi didn’t clearly show an improvement of calcium handling compared to the other ES groups, that can be due to the relatively short stimulation period (4 days) and the use of neonatal rat cardiomyocytes that limited the observable significant differences between groups. Finally, pharmacological blockade of connexins using carbenoxolone caused a significant increase in ET in the Asym Bi group, underscoring the functional importance of connexin-mediated coupling in this condition. Asym Bi also improved the cardiac maturation of 3D ECTs by enhancing the Actn2^+^ area of cardiomyocytes and the elongation of cardiomyocytes, and functionality by reducing the ET and enhancing the MCR compared to control. Moreover, contractility analysis revealed more reproducible and spatially uniform contractions in Asym Bi-stimulate ECTs, whereas Control and Mono groups showed higher variability and non-uniform contraction pattern. Overall, Asym Bi ES outperformed conventional monophasic ES while delivering lower total charge and energy to the cultured ECTs.

Future studies should investigate the applicability of Asym Bi stimulation to human-induced pluripotent stem cell-derived cardiomyocytes and explore its combination with native-like mechanical stimulation in cyclic stretch bioreactors^17,18,20^ or its integration into high-throughput platforms for drug screening or disease modeling. Additionally, optimization of stimulation parameters, such as progressively increasing the frequency over time, as demonstrated by Ronaldson-Bouchard et al.,^24^ may further enhance cardiac maturation and function. In the current study, the short-term culture setup did not allow for modulation of frequency or other parameters; however, future investigations with extended culture durations will enable these aspects to be explored. Moreover, future studies should also aim to achieve a deeper mechanistic understanding of the molecular and electrophysiological effects of the Asym Bi. In particular, pathway perturbation studies, single-cell analyses, including patch-clamp electrophysiology, will be essential to characterize ion channel activities, action potential properties, and excitation–contraction coupling at the cellular level. Finally, future studies should investigate protein-level changes directly within the 3D constructs to further confirm the benefits of the Asmy Bi stimulation.

In conclusion, this study demonstrates that Asym Bi significantly enhances the maturation and function of cardiomyocytes in both 2D and 3D* in vitro* culture models, while providing an optimal combination of total energy, total delivered charge and residual voltage. Asym Bi waveform outperforms conventional Mono and Sym Bi waveforms in energy efficiency, electrical functionality, contractility, calcium handling, metabolic maturity, and intercellular connectivity, offering a promising strategy for advancing cardiac tissue engineering platforms.

## Supplemental Material

sj-docx-1-tej-10.1177_20417314251393556 – Supplemental material for Asymmetric biphasic electric stimulation supports cardiac maturation and functionality Supplemental material, sj-docx-1-tej-10.1177_20417314251393556 for Asymmetric biphasic electric stimulation supports cardiac maturation and functionality by Antonio Sileo, Stefano Gabetti, Alp Can Gülan, Igor Cervenka, Chunyan Zhang, Alma Mingels, Giulia Milan, Diana Massai and Anna Marsano in Journal of Tissue Engineering
