# Supplementary material for: Asymmetric biphasic electric stimulation supports cardiac maturation and functionality
Source: J Tissue Eng. 2025 Nov 28;16:20417314251393556. doi: 10.1177/20417314251393556 (PMC12663056; doi:10.1177/20417314251393556)
Supplement: sj-docx-1-tej-10.1177_20417314251393556 – Supplemental material for Asymmetric biphasic electric stimulation supports cardiac maturation and functionality [file sj-docx-1-tej-10.1177_20417314251393556.docx]

Asymmetric biphasic electric stimulation supports cardiac maturation and functionality

*Antonio Sileo^1†^, Stefano Gabetti^1,2†^, Alp Can Gülan^1^, Igor Cervenka^3^, Chunyan Zhang^1^, Alma Mingels^4^, Giulia Milan^1^, Diana Massai^2^ and Anna Marsano^1^**

**Materials and methods Supplementary**

**Lumped-parameter model assumptions**

The three parameters of the Randles Cell (*R_e_*, *R_p_*, *C_p_*) were evaluated considering the materials and geometry of the chamber as described in previous publication^1^.

To determine *R_e_* we considered the conductivity of the solution σ and the geometry of the volume of electrolyte exposed to current flow. For an electrode area *A* facing the electrolyte and inducing a uniform current, and with *d* representing the spacing between the electrodes, the solution resistance is calculated as follows ^2^:

$$R_{e}=\frac{d}{\sigma A}$$

where *σ* is the conductivity of the solution and *A* is the area of the electrode exposed to the electrolyte.

The value of *A* was calculated by assuming that the cylindrical carbon rod electrodes in the chamber expose two-thirds of their lateral surface area to the electrolyte:

$$A=2\pi\cdot r\cdot l\cdot\frac{2}{3}$$

Considering the length of the electrode portion exposed to the electrolyte (*l* = 20 mm), the electrode radius (*r* = 1.5 mm), and the interelectrode distance (*d* = 1 cm), the resulting area was *A* = 1.26 cm^2^.

Using the conductivity value of the culture media reported in the literature (1.5 S/m ^3^), *R_e_* was estimated to be 53 Ω.

The polarization resistance *R_p_* and the double-layer capacitance *C_p_* were evaluated based on literature data from electrochemical impedance spectroscopy (EIS) studies which provided normalized values per unit area (*R_p_/A*= 4.06*10^13^ Ω/cm^2^; *C_p_/A*= 190 µF/cm^2^ ^4^). This resulted in *R_p_* = 5.13 x 10^8^ MΩ and *C_p_* = 240 µF.

**References**

1. Gabetti S, Sileo A, Montrone F, et al. Versatile electrical stimulator for cardiac tissue engineering—Investigation of charge-balanced monophasic and biphasic electrical stimulations. *Front Bioeng Biotechnol*; 10, https://www.frontiersin.org/articles/10.3389/fbioe.2022.1031183 (2023, accessed 4 January 2023).

2. Tandon N, Marsano A, Cannizzaro C, et al. Design of electrical stimulation bioreactors for cardiac tissue engineering. In: *2008 30th Annual International Conference of the IEEE Engineering in Medicine and Biology Society*. Vancouver, BC: IEEE, pp. 3594–3597.

3. Tandon N, Marsano A, Maidhof R, et al. Optimization of electrical stimulation parameters for cardiac tissue engineering. *J Tissue Eng Regen Med* 2011; 5: e115–e125.

4. Tandon N, Cannizzaro C, Figallo E, et al. Characterization of Electrical Stimulation Electrodes for Cardiac Tissue Engineering. In: *2006 International Conference of the IEEE Engineering in Medicine and Biology Society*. New York, NY: IEEE, pp. 845–848.

**Supplementary Figure S1. Comparison of the theoretical ES modes used in biological experiments**. The absolute value electric field variation (orange bar) is 3 V/cm for both the Monophasic ES (left) at 3 V/cm and the Symmetric Biphasic ES at ± 1.5 V/cm (center). In contrast, the Asymmetric Biphasic ES at +3 / -1 V/cm (right) has a variation of 4 V/cm.

**

**Supplementary Figure S2. Effects of ES on ECT maturation**. A) Fibrin gel-based 3D construct placed on a PDMS membrane within a custom culture chamber, B) culture chambers positioned in a 6-well plate, each containing a fibrin gel-based construct.

**

**Supplementary Figure S3. Comparison of the experimental ES modes used in biological experiments.** Experimental waveforms for Monophasic (Mono) ES (top), Symmetric Biphasic (Sym Bi) ES (center) and Asymmetric Biphasic (Asym Bi) ES (bottom). The total charge delivered (indicated by the orange area) is highest for Mono ES at 3 V/cm, which delivers twice the charge of Sym Bi ES at ± 1.5 V/cm. The charge delivered by Asym Bi ES at +3/ -1 V/cm is intermediate between the two.

**Supplementary Figure S4. Experimental Voltage measurement.** Voltage measured across the sensing resistor for Mono ES (top), Sym Bi ES (center), and Asym Bi ES (bottom). The residual voltage (highlighted by the orange circle) for Mono ES at 3 V/cm is the most negative, indicating significant charge accumulation at the electrode-electrolyte interface. In contrast, the residual voltage for Sym Bi ES (± 1.5 V/cm) and Asym Bi ES at (+3/ -1 V/cm) are close to 0 V, suggesting minimal charge buildup.

**Supplementary Figure S5. Heatmap of the 10% most variable proteins for the experimental groups (Control, Mono, Sym Bi and Asym Bi).** Each row represents a single protein. Each column represents an individual sample. Protein abundance values are represented as z-scores, calculated per protein across samples, to highlight relative expression patterns; high expression is shown in red, low expression is shown in blue.

**

**Supplementary Figure S6. Effects of electrical stimulation on cell viability and damage-related protein expression.** Heatmap showing the relative expression of proteins associated with cell damage and cell death, based on z–score–normalized proteomic data. Color intensity reflects the average relative expression level across experimental groups: Control (n= 4 replicates), Mono (n= 4 replicates), Sym Bi (n= 3 replicates), and Asym Bi (n= 5 replicates). Each row represents a single protein. Higher expression levels are shown in red; lower expression levels are shown in blue.

**Supplementary Figure S7. Pairwise comparisons of differentially expressed proteins and GO: BP enrichment analyses.** Pairwise comparisons of Mono vs Control (A-B), Sym Bi vs Control (C-D), and Sym Bi vs Mono (E-F) are shown. In the volcano plots (A, C, E), the x-axis represents log_2_(fold change) and the y-axis represents -log_10_ (p-value) for the differential expression analysis. In the upper right corner, the number of proteins increased and in the upper left corner, the number of proteins decreased. Significance was determined based on the adjusted p-value (FDR<0.05) and shown as a vertical grey dotted line. Red and blue dots indicate proteins with significantly increased or decreased abundance, respectively, while gray dots represent proteins without significant changes. Protein names outlined in red or blue denote significant increases or decreases in protein levels, respectively. Proteins are grouped into 4 categories based on their function: blue (death and survival), pink (homeostasis), orange (metabolism), and green (sarcomere structure and contractility). GO:BP enrichment analysis results (B, D, F) are plotted with the x-axis indicating log_2_ (fold change). Pathways with a false discovery rate (FDR) below 0.1 were considered significantly enriched

****Supplementary Figure S8. Effects of electrical stimulation on cell damage**. A) Cardiomyocyte damage and B) Fibroblasts damage analysis. Early apoptotic cells were identified as Cleaved-Casp3^+^ and EthD-1^-^ (Control= 8 replicates, Mono= 7 replicates, Sym Bi= 8 replicates, Asym Bi= 8 replicates), apoptotic death as Cleaved-Casp3^+^ and EthD-1^+^ (Control= 8 replicates, Mono= 7 replicates, Sym Bi= 8 replicates, Asym Bi= 8 replicates), non-apoptotic dead as Cleaved-Casp3^-^ and EthD-1^+^ (Control= 10 replicate, Mono= 9 replicates, Sym Bi= 10 replicates, Asym Bi= 10 replicates). Cardiomyocytes were identified as Actn2^+^ and DAPI^+^. Fibroblasts were identified as Actn2^-^ and DAPI^+^. C) Ratio cTnI/cTnT at the end of the3 days of preculture (n= 16 replicates) and cultured for 5 and 7 days under Control (n= 8 replicates), Mono (n= 8 replicates), Sym Bi (n= 8 replicates) and Asym Bi (n= 8 replicates). G) ROS quantification in supernatants at the end of the 7 days of culture for the Control (n= 4 replicates) and the electrically stimulated groups Mono (n= 4 replicates), Sym Bi (n= 4 replicates) and Asym Bi (n= 4 replicates). E) Cell viability with culture medium exposed to 2 days under the experimental conditions used for electrical stimulation (Mono= 4 replicates, Sym Bi= 4 replicates, Asym Bi= 4 replicates) normalized to the Control group. Statistical analysis was performed using a nonparametric Kruskal-Wallis test.

**

**Supplementary Figure S9. Effects of electrical stimulation on cardiac maturation**. A) Sarcomere length quantification for the Control (n= 22 replicates), Mono (n= 19 replicates), Sym Bi (n= 18 replicates), and Asym Bi (n= 20 replicates). B) Distribution of sarcomere length quantification in different bins for the Control (n= 22 replicates), Mono (n= 19 replicates), Sym Bi (n= 18 replicates), and Asym Bi (n= 20 replicates). C) Percentage of area Actn2^+^ normalized by the number of CMs for the electrically stimulated groups Mono (n= 19 replicates), Sym Bi (n= 18 replicates), and Asym Bi (n= 20 replicates). D) Fold increase of the percentage of area Cx-43^+^ normalized to the number of CMs for the electrically stimulated groups Mono (n= 9 replicates), Sym Bi (n= 6 replicates) and Asym Bi (n= 9 replicates). Statistical analysis was performed using A) one-way ANOVA test, B) Pairwise Fisher’s exact tests, C) nonparametric Kruskal-Wallis test and D) nonparametric Kruskal-Wallis test. Asterisks (*) denote statistical significance (*p < 0.05, **p < 0.01, ***p < 0.001).

**Supplementary Figure S10. Movie analysis for cardiomyocyte contractility assessment.** Displacement magnitudes of consecutive contractions of CMs for the different culture conditions. A) Control, B) Mono, C) Sym Bi and D) Asym Bi.

***Supplementary Video S1. Fluo-4 fluorescence video of the Control group*** ***of CMs cultured for 7 days without ES.***

***Supplementary Video S2. Fluo-4 fluorescence video of the Mono group*** ***of CMs cultured for 3 days without ES + 4 days with 3 V/cm Mono ES.***

***Supplementary Video S3. Fluo-4 fluorescence video of the Sym Bi group*** ***of CMs cultured for 3 days without ES + 4 days with ± 1.5 V/cm Sym Bi ES.***

***Supplementary Video S4. Fluo-4 fluorescence video of the Asym Bi group*** ***of CMs cultured for 3 days without ES + 4 days with +3/ -1 V/cm Asym Bi ES.***

***Supplementary Video S5. Contraction of cardiomyocytes cultured without electrical stimulation (Control).***

***Supplementary Video S6. Contraction of cardiomyocytes cultured with Mono.***

***Supplementary Video S7. Contraction of cardiomyocytes cultured with Sym Bi.***

***Supplementary Video S8. Contraction of cardiomyocytes cultured with Asym Bi.***

***Supplementary Video S9. Control ECT strain heat maps video during the contraction.***

***Supplementary Video S10. Mono ECT strain heat maps video during the contraction.***

***Supplementary Video S11. Asym Bi ECT strain heat maps video during the contraction.***
